# Assessing quality standards for ChIP-seq and related massive parallel sequencing-generated datasets: When rating goes beyond avoiding the crisis

**DOI:** 10.1016/j.gdata.2014.08.002

**Published:** 2014-08-14

**Authors:** Marco Antonio Mendoza-Parra, Hinrich Gronemeyer

**Affiliations:** Department of Functional Genomics and Cancer, Institut de Génétique et de Biologie Moléculaire et Cellulaire, Equipe Labellisée Ligue Contre le Cancer, Centre National de la Recherche Scientifique UMR 7104, Institut National de la Santé et de la Recherche Médicale U964, University of Strasbourg, Illkirch, France

**Keywords:** ChIP sequencing, Massive parallel sequencing, Quality control, Omics data mining

## Abstract

Massive parallel DNA sequencing combined with chromatin immunoprecipitation and a large variety of DNA/RNA-enrichment methodologies is at the origin of data resources of major importance. Indeed these resources, available for multiple genomes, represent the most comprehensive catalogue of (i) cell, development and signal transduction-specified patterns of binding sites for transcription factors (‘cistromes’) and for transcription and chromatin modifying machineries and (ii) the patterns of specific local post-translational modifications of histones and DNA (‘epigenome’) or of regulatory chromatin binding factors. In addition, (iii) the resources specifying chromatin structure alterations are emerging. Importantly, these types of “omics” datasets populate increasingly public repositories and provide highly valuable resources for the exploration of general principles of cell function in a multi-dimensional genome–transcriptome–epigenome–chromatin structure context. However, data mining is critically dependent on the data quality, an issue that, surprisingly, is still largely ignored by scientists and well-financed consortia, data repositories and scientific journals. So what determines the quality of ChIP-seq experiments and the datasets generated therefrom and what refrains scientists from associating quality criteria to their data? In this ‘opinion’ we trace the various parameters that influence the quality of this type of datasets, as well as the computational efforts that were made until now to qualify them. Moreover, we describe a universal quality control (QC) certification approach that provides a quality rating for ChIP-seq and enrichment-related assays. The corresponding QC tool and a regularly updated database, from which at present the quality parameters of more than 8000 datasets can be retrieved, are freely accessible at www.ngs-qc.org.

With the release of the first draft of the human genome in 2001 [1], [2] and the continued dramatic development of massive parallel sequencing technologies, the race is open towards the analysis of genome functions from a holistic point of view and a rapidly increasing fraction of the medico-scientific community is getting engaged in this endeavor. Notably, the combination of high-throughput massive parallel sequencing technology with a molecular biology technique termed chromatin immunoprecipitation (ChIP), which was first described 30 years ago [3], provided an increasingly affordable high resolution approach to interrogate protein interactions with entire genomes. In fact, in 2007 three publications described for the first time the use of massive parallel sequencing for global mapping of several factors to chromatin [4], [5], [6] and coined the term “ChIP-seq” in analogy to the earlier ChIP-chip technology, which involved the use of DNA microarray chips probed with ChIPed DNA [7].

In the following seven years, more than 1300 publications related to the term “ChIP-seq” were indexed in MEDLINE, covering a large range of evaluated genome interactors, including not only transcription factors, but also several post-translational modifications of histones (termed histone ‘marks’), which together with DNA modifications define the functional organization of the epigenome. In addition, a variety of DNA/RNA-enrichment methodologies have been combined with massive parallel sequencing, which together with ChIP-seq assays, the resulting datasets populate increasingly public repositories like the Gene Expression Omnibus (GEO). Importantly, these resources are of major interest as they represent the most comprehensive catalogue of global protein–chromatin interaction, chromatin modification patterns and chromatin architecture for several genomes, inviting for genome-wide multi-profile comparisons in silico.

One of the major challenges in the present “big data” era is to design computational solutions that extract meaningful information from the huge amounts of available data. In the “omics world” and more specifically in the ChIP-seq and enrichment-related data resources, the aim is (i) to discern significant enrichment events from background noise present in each studied profile and (ii) to perform multiprofile comparisons in the context of their enrichment patterns to infer co-occurring events that are the basis or consequence of, or linked to the biological functions under study. In fact, since the release of the first ChIP-seq datasets, multiple computational solutions were developed for the mapping of enrichment events in a confident manner and several recent studies focused on multiprofile comparisons (for a detailed review see [8]). However, one of the pre-requisites for any comparison is that the compared items are indeed comparable. Applied to ChIP-seq this implies that only datasets of similar quality can be compared. But what determines the quality of ChIP-seq experiments and the datasets generated therefrom?

Assessing the quality of ChIP-seq datasets requires evaluation of the performance of all involved steps — from the chromatin immunoprecipitation up to the construction of the sequencing library. In fact, while the generally certified sequencing procedure itself is nowadays of very high quality and reliability, the quality issues arise at the earlier steps of a ChIP-seq procedure. Aspects like antibody specificity and sensitivity, crosslinking and optimal chromatin fragmentation, number of PCR amplification rounds applied during preparation of the sequencing library, as well as the number of sequenced DNA molecules (also referred to as sequencing depth) per assay have a direct impact on the quality of the generated dataset. While in principle each of these steps might follow its own quality standards, there are important differences in the way different laboratories handle the quality of antibodies or the minimal sequencing depth required for generating optimal ChIP-seq datasets.

To address this issue, several recent studies have described different types of quality metrics that would complement the visual inspection in a genome browser - commonly performed as an intuitive way to evaluate the quality of a ChIP-seq assay - with quantitative approaches. Among them, the ENCODE consortium described recently guidelines for ChIP-seq assays [9], which not only cover the experimental design but also propose a certain number of computational metrics for the evaluation of the quality of ChIP-seq generated datasets. However, while these recommendations may be useful to improve the quality of future datasets, each of these metrics has limitations, as discussed in [10].

Overall, two major principle methodologies have been described to generate such metrics, (i) those based on the use of peak caller *algorithms* as initial read-outs and (ii) those assessing quality descriptors in a peak caller-independent manner. The first category corresponds to methodologies which assess, for example, the Fraction of Reads in Peaks [FRIP] [9] or the Irreproductibility Discovery Rate [IDR] [9], [11] from the comparative analysis of ChIP-seq replicates. In both cases the need of using peak calling algorithms prior to quality evaluation is a major drawback, as a large variety of peak calling methods perform with high variability and in addition they operate with user-defined parameters. Furthermore, depending on the nature of the enrichment type (e.g., sharp binding events or broad enrichment patterns) different peak callers are required for optimal identification of enrichment patterns. This makes it very difficult, and frequently impossible to compare such quality metrics when performing multiprofile comparisons. Furthermore, the quality assessment by comparing ChIP-seq replicates like that performed in IDR can only provide information about the reproducible fraction of peaks shared between two replicate datasets, but it will not distinguish per se the quality of the two individual datasets. In addition, IDR is based on the availability of at least two replicates per assay, which is generally suggested but (for cost reasons) often not respected. This is confirmed by our evaluation of available datasets in the GEO repository. Finally, it is worth mentioning that a comparison of multiple ChIP-seq replicates per definition reveals the degree of reproducibility of identified enrichment events but reproducibility does not automatically mean quality. For instance, multiple replicates produced at suboptimal sequencing depths may suggest a low reproducibility, albeit the corresponding chromatin immunoprecipitation may be of high quality. In such cases combining all replicates in a single dataset followed by sub-sampling of mapped reads (see below) could tell whether the sequencing depth used accounts indeed for low pattern reproducibility, or whether this is rather the consequence of a poor ChIP assay.

As part of the second category, we could cite for instance the strand-cross correlation approach [12] which computes the asymmetry between forward and reverse strands. Importantly, while this approach is applicable to profiles with sharp peaks, such as those seen for transcription factors, it cannot be used for the broad profiles often seen for histone marks. Thus, the absence of a general quality assessment for the large amount of ChIP-seq datasets currently available in the public domain, which allows their direct comparison, is a major handicap for performing optimal multiprofile in silico data analysis.

With the aim of assessing quality descriptors in a peak-caller independent manner, we have developed a quality control (QC) metrics applicable to any kind of dataset generated by massive parallel DNA sequencing from ChIP-seq and other DNA/RNA enrichment-based technologies [10]. Briefly, this QC assessment compares the enrichment patterns retrieved in a given profile when only a fraction of the total mapped reads is used for its reconstruction and provides local (i.e. related to a defined genomic region) and global (i.e. related to the whole dataset) QC indicators, which for simplicity are also represented by a 3-letter acronym or Quality Stamps,^1^ similar to that used by credit rating agencies (‘AAA’ for best quality datasets to ‘DDD’ for worst; see Fig. 1). Without going into the details concerning its functionality, this methodology overcomes the above-described problems, making it a universal approach for assessing and comparing the quality of enrichment-based sequencing datasets. Note that the QC descriptors of more than 8000 publicly available datasets have been evaluated at present; we are currently generating QC data for virtually all publicly available and newly generated datasets using a fully automated procedure. This certification database (see Fig. 2 for a screenshot) is freely accessible through a dedicated website (www.ngs-qc.org). This database is the most comprehensive numerical QC collection for enrichment-based NGS profiles and due to its “universal” application, it provides a solid ground for multiprofile comparisons.

Publicly available ChIP-seq datasets have been generated from platforms with a wide range of sequencing capacity, ranging from less than 5 million sequenced reads in the early times of the first Illumina Genome Analysers to more than hundred million reads per dataset/sample from a single lane when using the present sequencer generations. Despite this technological progress low quality datasets are still retrieved at this point in time, arguing that inappropriate sequencing depths have been chosen due to the possibility of saving costs by multiplexing and/or that factors other than the sequencing depth are causing bad performance (Fig. 3A). Nevertheless, as illustrated in Fig. 3B, it is now possible to extract information about the minimal sequencing depth that should be used for a given factor from the important number of datasets available.

Another parameter that directly influences the quality of ChIP-seq assays is the antibody used. Unfortunately, with the advent of the ‘omics’ generation, the commercial market for antibodies got swamped with pseudo grades describing an antibody as “ChIP grade”, “ChIP-chip grade” or “ChIP-seq” grade. While ChIP grades are generally accompanied by quantitative real-time PCR assays to support this statement, ChIP-chip and ChIP-seq grades are only supported by local enrichment patterns displayed in genomic screenshots without providing quantitative measure for such grading. In addition, some vendors support their grading by making reference to a (prestigious) peer-reviewed publication where ChIP-seq or ChIP-chip assays have been performed with this antibody, without considering whether that publication is based on high or low quality enrichment patterns. It is clear that, despite the existence of a set of quality descriptors, the use of the term “ChIP-seq grade” is rather a marketing strategy than a real source of information. Indeed, what is required is an independent QC assessment procedure that applies defined ChIP procedures and QC metrics to characterize the performance of an antibody in ChIP-seq and related assays. At this respect, it is worth to mention that the use of our methodology [10] for assessing the quality of publicly available datasets allows extrapolating the performance of the antibodies in use and by consequence to identify potential “ChIP-seq grade” antibodies this time by applying a quantitative and independent certification procedure.

In summary, the bad news is that despite the fast progress in the ‘omics’ field still many low quality ChIP-seq and enrichment-based datasets are generated and that there is no requirement to ascribe a quality indicator to ChIP-seq datasets at publication. The good news is that the large amount of datasets released in the public domain represents a highly valuable resource for mining the quality of the evaluated datasets. We note, however, that unfortunately not all of the publicly well-funded consortia provide access to their raw data, such that their quality can be evaluated, neither systematically associates to their profiles a given quality assessment. For this reason, we have developed the NGS-QC system, which aids in extrapolating quality guidelines from publicly available datasets and thereby hopefully influences the quality of future datasets. Indeed, the NGS-QC descriptors represent for the first time a universal rating to classify datasets and antibodies.

It is very obvious from the NGS-QC database (www.ngs-qc.org) that there is a large variation in the quality of ChIP-seq and other enrichment-based NGS datasets. Several lines of arguments support our view that future enrichment-based NGS datasets should be accompanied by quantitative QC metrics: (i) Given the investment in time, effort and (public) costs of these assays, they deserve a quality stamp that gives confidence to other colleagues for using these datasets in *meta*-analyses and considering them good enough to draw conclusions in the context of their own studies; this way unnecessary and costly repetitions of the same assays can be minimized; (ii) currently it is nearly impossible for editors, reviewers and readers of a study to assess the quality of a ChIP-seq profile, since screenshots do definitely not reveal the quality of an entire ChIP-seq profile. The addition of QC metrics to a profile will enhance both the confidence in, and reproducibility of the data; (iii) the development of novel types of epi-drugs and the increasingly widespread use of enrichment-based NGS technologies in drug development involves sooner or later the regulatory authorities to request quality assessment metrics for these data. It is thus wise that the scientific community adopts a generally applicable QC assessment procedure and establishes guidelines that associates such metrics with all published datasets; (iv) in the near future the development of personalized medicine and the cost reductions from 4th generation sequencing will result in a vast increase in the number of ‘omics’ datasets, including ChIP-seq and alike. The scientific community needs to be prepared to distinguish high quality profiles from rubbish in order to protect the consumer — the clinician/patient or any authorized user who is generally not familiar with the interpretation of such large datasets and the corresponding profiles.

Considering the importance of the quality assessment of enrichment-based NGS datasets for the scientific community, the responsibility towards granting institutions who invest enormous amounts of money in a large number of consortia using these technologies, and to the public that has the right to ask for an investment that generates highest quality data, we propose that each dataset should be accompanied by a quality assessment. We suggest that data repositories, such as GEO, and journals ask for data quality assessment before they make new datasets publicly available. The risk that we are facing is not only that low quality data are filling up data repositories, which are or will be soon short of storage space, but also that the extraction of highly valuable information from already existing data will be precluded by their incomparability due to low quality.

## Conflict of interest

None declared.

## Figures and Tables

**Fig. 1:**
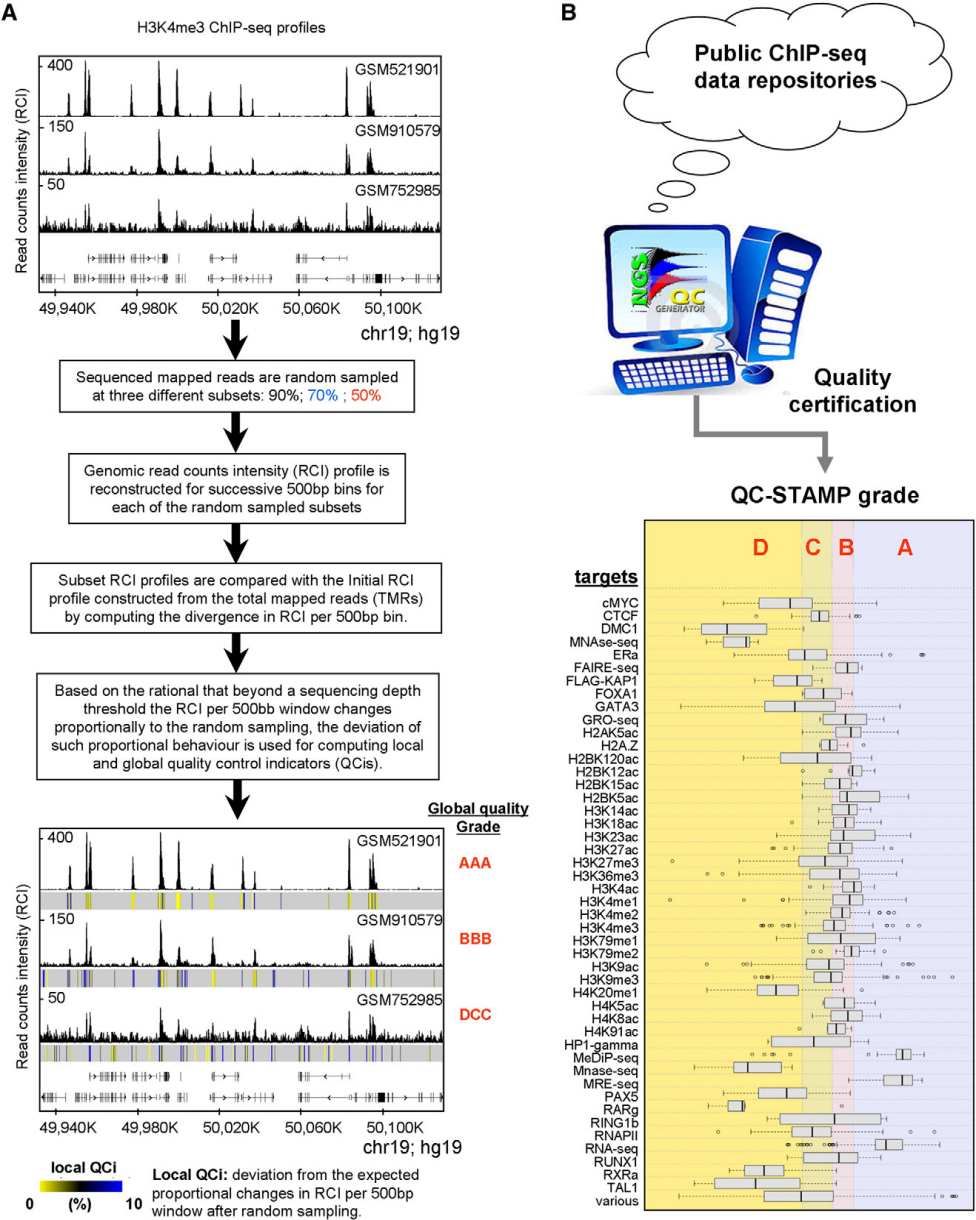
Strategy applied by the NGS-QC Generator for assessing quality descriptors for ChIP-seq profiles. (A) Genome-browser screenshot illustrating three publicly available H3K4me3 ChIP-seq datasets. Based on a visual inspection we could conclude that, while all three profiles share common binding sites, important differences in their read count intensity levels as well as in their background noise are observed. These datasets were subjected to the NGS-QC Generator pipeline for assessing quality descriptors. Briefly, TMRs are randomly sampled into three distinct populations (90, 70 and 50%), which are used for profile reconstruction by computing the RCIs in 500-bp bins. The RCI divergence from expectation is measured relative to the original profile (s100). This information generates local quality indicators (QCis) and is displayed together with the original RCI profile to identify robust chromatin regions (local QCi heat-map below the bottom profiles). In addition, global quality descriptors are computed which are summarized into the Global quality Grade or QC-STAMP. In this particular example, the best H3K4me3 dataset received a “AAA” grade while the worst has been discerned a “DCC” QC-STAMP. It is worth to mention that the intuitive quality assessment performed by the visual inspection is now comforted by a global and quantitative QC descriptor. (B) The NGS-QC Generator has been used to perform quality certification of datasets retrieved in publicly available repositories. Currently, more than 8000 datasets were certified covering a variety of data types and were classified based on a quality score grades from AAA for the highest quality datasets to DDD for those presenting the worst, like input control datasets.

**Fig. 2:**
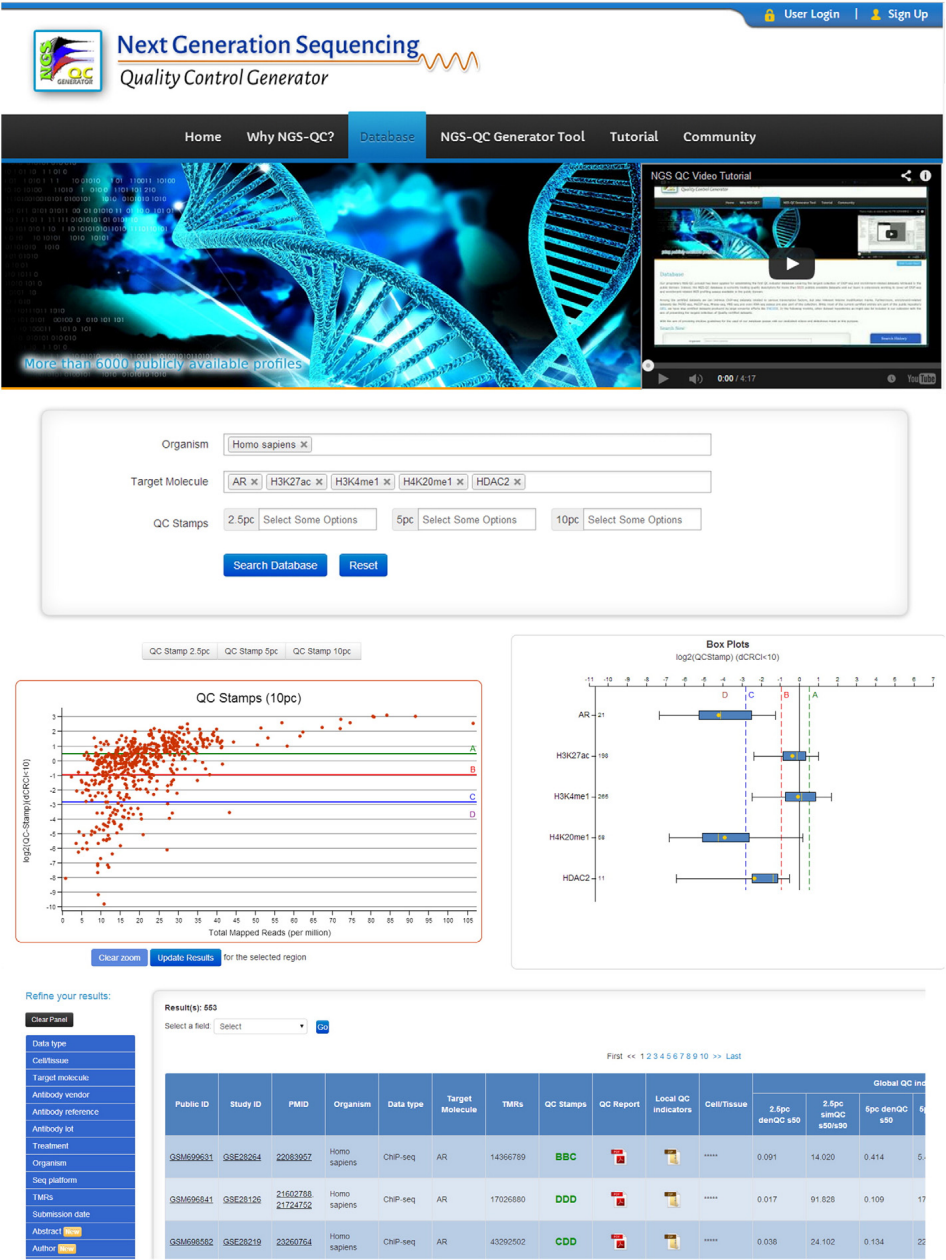
Screenshot (www.ngs-qc.org) of a search for ChIP-seq profiles for 5 targets (androgen receptor, AR; H3K27ac; H3K4me1; H4K20me1; HDAC2) choosing as organism “*Homo sapiens*”. A scatter plot (middle left) shows the different QC Stamps and indicates the quartiles (A, B, C, D) and the corresponding read depths using 10% divergence from expectance. The box-plot on the right reveals the corresponding range of QC Stamps for each target. Details of each dataset are given at the bottom. In addition, Abstracts of associated publications to the displayed datasets are also available as a pop up window in our website (not shown) as well as a powerful search refining panel. For details see the online manual and reference [10].

**Fig. 3:**
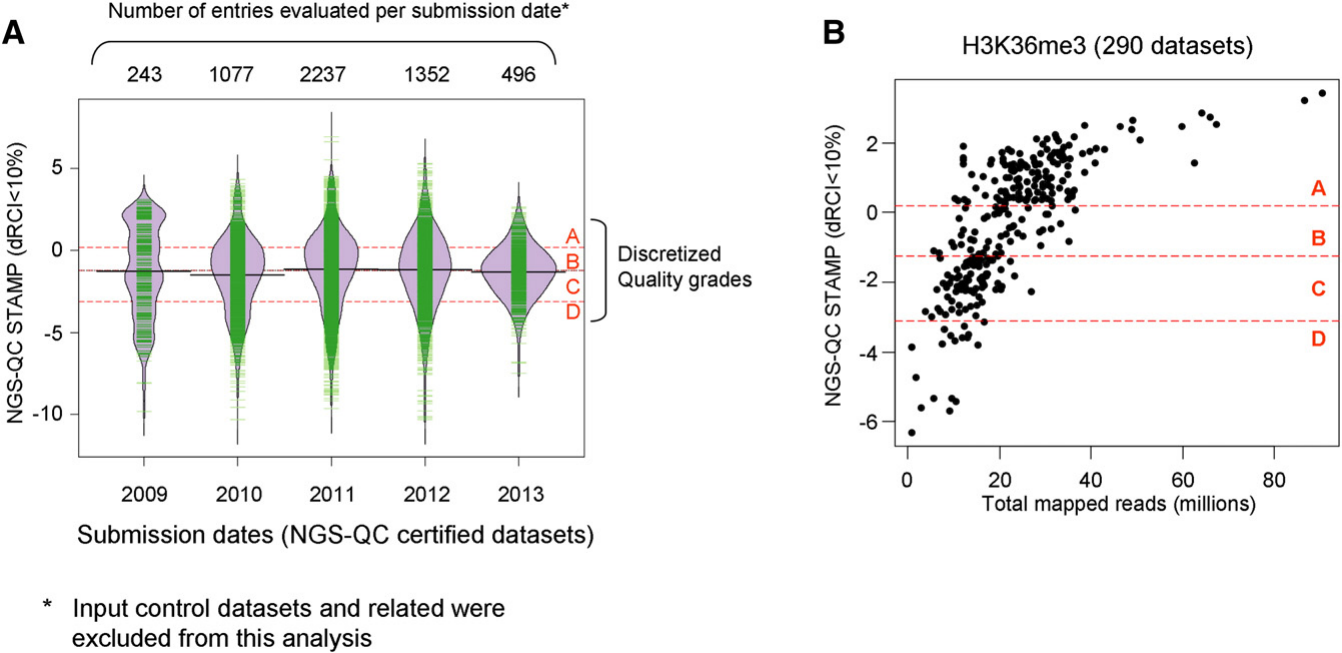
(A) NGS-QC certified datasets classified in the context of their repository submission dates. The illustrated violin plots are complemented by individual observations (green lines) to enhance their density over different NGS-QC quality grades (grade borders are illustrated by red dashed lines). Note that input control datasets were excluded from this classification to avoid an artificial low quality bias. (B) Scatter-plot illustrating the correlation between the total mapped reads per analyzed H3K36me3 datasets and their quality indicator (NGS-QC STAMP). Note that the majority of datasets presenting less than 15 million TMRs are associated with quality grades “B”, “C” or “D”. In fact, quality “A” datasets are preferentially associated to TMRs higher than 15 million reads.

## Footnotes

1 The NGS-QC Quality Stamp is a simplified quality assessment that describes the quality of a profile relative to the distribution of the QC indicators in the database. It is defined as QC-STAMP = denQCi(s50) / simQCi, where denQCi(s50) (or density quality control indicator or QCi) is the fraction of genomic regions presenting a proportional decrease (under defined dispersion intervals) of their related read counts when 50% of total mapped reads (TMRs) are used for profile reconstruction and simQCi (or similarity QCi) corresponds to the ratio between the denQCi computed for 90% TMRs over that computed for 50% TMRs. Intuitively, the higher the denQCi at 50% TMRs (s50) and lower the simQCi (i.e. less difference between the denQCi at 90 and 50% TMRs), the higher the QC-STAMP descriptor. The proportional decrease is computed for three dispersion interval thresholds, namely 2.5%, 5% and 10%; thus three QC-STAMP values are computed per evaluated profile. As mentioned above, the comparison of these 3 QC-STAMPs with those computed over the whole NGS-QC database provides a direct way to express the quality of a given dataset relative to the whole database. Specifically, the QC-STAMPs in the database are divided into four quartiles, each of them represented by a grade (from A to D associated with the upper to lower quartile respectively). Thus a dataset described as “AAA” presents each of the 3 QC-STAMPs (2.5%, 5% and 10% dispersion) as part of the upper quartile of the quality descriptors computed from the NGS-QC database.
